# Supplementary material for: Influence of Tethered Ions on Electric Polarization and Electrorheological Property of Polymerized Ionic Liquids
Source: Molecules. 2020 Jun 23;25(12):2896. doi: 10.3390/molecules25122896 (PMC7356505; doi:10.3390/molecules25122896)
Supplement: Supplementary file 1 [file molecules-25-02896-s001.pdf]

Supplementary Information

Influence of Tethered Ions on Electric Polarization and Electrorheological Property of Polymerized Ionic Liquids

Fang He, Bo Wang, Jia Zhao, Xiaopeng Zhao, Jianbo Yin*

Smart Materials Laboratory, Department of Applied Physics, Northwestern Polytechnical University, Xi'an, 710129, People's Republic of China; xgdhefang@mail.nwpu.edu.cn (F.H.); wangbo0304@iccas.ac.cn (B.W.); zhaojia11@mail.nwpu.edu.cn (J.Z.); xpzhao@nwpu.edu.cn (X.P.Z.)

* Corresponding author: jbyin@nwpu.edu.cn

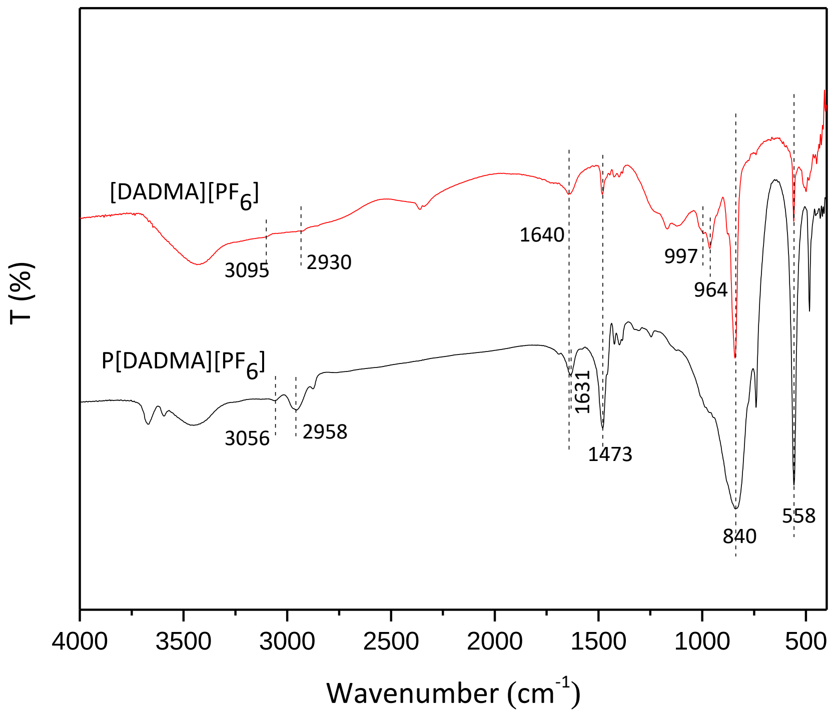

Figure S1. FT-IR spectra of [DADMA][PF_6] and P[DADMA][PF_6].

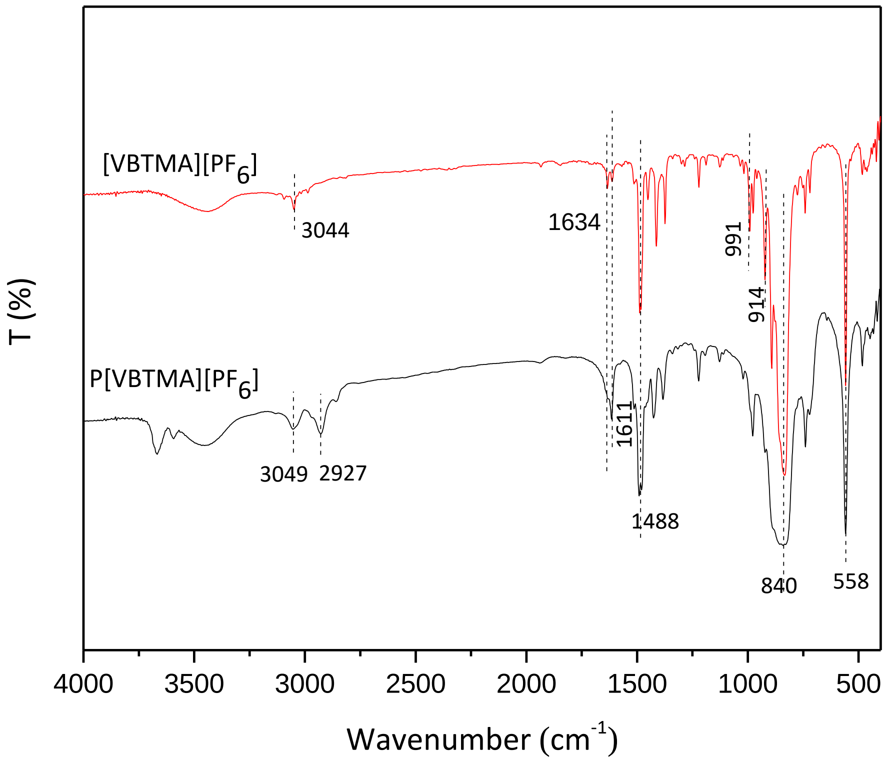

Figure S2. FT-IR spectra of [VBTMA][PF_6] and P[VBTMA][PF_6].

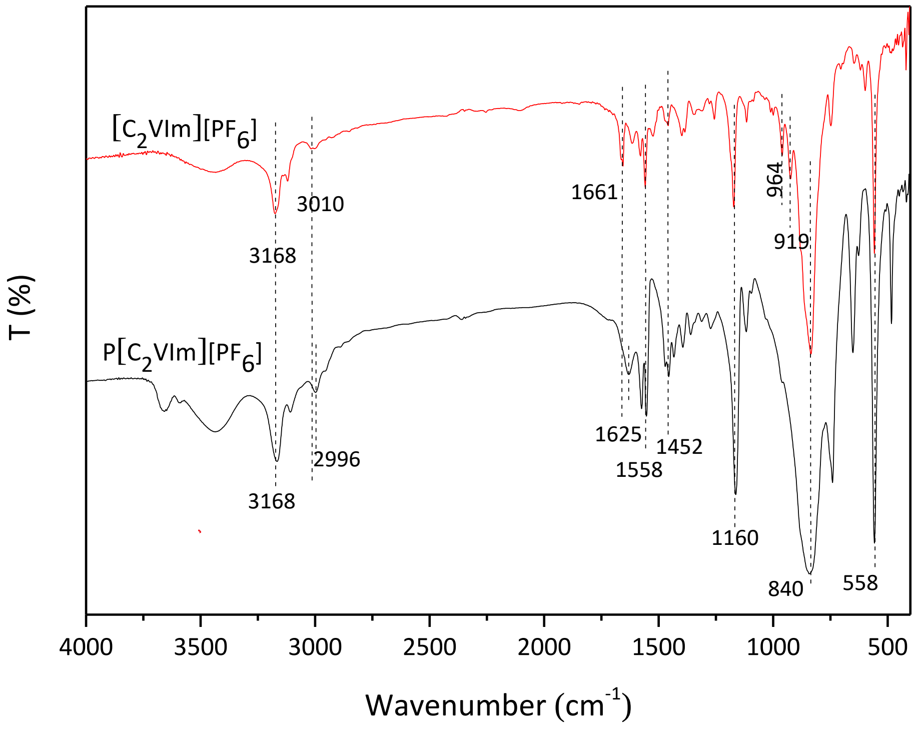

Figure S3. FT-IR spectra of $[C_2VIm][PF_6]$ and $P[C_2VIm][PF_6]$.
